# Chromosomal and Plasmid-Based CRISPRi Platforms for Conditional Gene Silencing in *Lactococcus lactis*

**DOI:** 10.3390/ijms26199516

**Published:** 2025-09-29

**Authors:** Chenxi Huang, Meishan Liu, Jan Kok

**Affiliations:** 1 Key Laboratory of Combinatorial Biosynthesis and Drug Discovery (Ministry of Education), School of Pharmaceutical Sciences, Wuhan University, Wuhan 430071, China; 2 Department of Molecular Genetics, Groningen Biomolecular Sciences and Biotechnology Institute, University of Groningen, 9747AG Groningen, The Netherlands; meeishaan.liu@gmail.com

**Keywords:** CRISPRi, *Lactococcus lactis*, gene silencing, YbeY

## Abstract

Inducible CRISPR interference (CRISPRi) systems were established in *Lactococcus lactis* using both plasmid and chromosomal approaches. Expression of nuclease-deficient Cas9 (dCas9) from *Streptococcus pyogenes* was placed under the control of the nisin-inducible promoter P_nisA_, while sgRNAs were transcribed from the constitutive P_usp45_ promoter. To monitor expression, dCas9 was fused with superfolder GFP. Plasmid-based constructs successfully repressed a luciferase reporter gene and silenced the gene of the major autolysin, AcmA, leading to the expected morphological phenotype. However, plasmid systems showed leaky expression, producing mutant phenotypes even without induction. Chromosomal integration of *dCas9* reduced its expression level by approximately 20-fold compared with plasmid-based expression, thereby preventing leaky activity and ensuring tight regulation. This chromosome-based (cbCRISPRi) platform enabled controlled repression of the essential gene *ybeY*, which resulted in severe growth defects. Restoration of wild-type phenotypes was achieved by introducing a synonymous codon substitution in the sgRNA target region. Transcriptome analysis of *ybeY*-silenced cells revealed downregulation of ribosomal protein genes and widespread effects on membrane-associated proteins, ATP synthase subunits, and various transporters. These inducible CRISPRi platforms provide robust and tunable tools for functional genomics in *L. lactis*, particularly for studying essential genes that cannot be deleted.

## 1. Introduction

*Lactococcus lactis* is one of the most extensively studied lactic acid bacteria (LAB) and plays a central role in the dairy industry as a starter culture [1]. Beyond its traditional application, this species has been widely engineered as a microbial cell factory for producing metabolites such as mannitol and lactic acid [2], as a host for recombinant protein production [3], and as a delivery vehicle for vaccines and therapeutic proteins [4]. These applications have been facilitated by the development of a versatile set of genetic engineering tools. Several inducible systems have been explored in *L. lactis*, including zinc-inducible, low-pH-inducible, agmatine-inducible, and nisin-inducible promoters [5]. Among the available systems, the nisin-controlled expression (NICE) platform is the best-characterized and most commonly used strategy for inducible gene expression in *L. lactis*. [6]. NICE relies on the two-component regulatory system NisK–NisR, which responds to the lantibiotic peptide nisin, a natural product of some *L. lactis* strains [7].

For genome modification, methods such as double-crossover recombination using non-replicative vectors (e.g., pORI [8] or pCS1966 [9]) are available, but generating stable mutants typically requires several weeks, highlighting the need for faster and more flexible alternatives. The CRISPR-Cas system (clustered regularly interspaced short palindromic repeats and CRISPR-associated proteins) constitutes an RNA-guided adaptive immune defense in bacteria and archaea, where it protects cells from invading genetic elements such as phages and plasmids [10]. In the widely used *Streptococcus pyogenes* system, Cas9 functions as a DNA endonuclease directed by an RNA duplex that base-pairs with a complementary DNA sequence, generating a double-strand break (DSB) precisely at the target locus [11]. This duplex consists of a CRISPR RNA (crRNA), whose maturation requires RNase III cleavage, and a trans-activating RNA (tracrRNA). To simplify the system, a chimeric single-guide RNA (sgRNA) was designed to combine the roles of crRNA and tracrRNA, thereby avoiding the need for RNase III processing [12].

For gene repression, a catalytically inactive variant of Cas9 (dCas9) can be employed in CRISPR interference (CRISPRi). Guided by an sgRNA, dCas9 binds to a selected genomic locus—commonly near a promoter or within a coding sequence—where it obstructs RNA polymerase binding or transcript elongation, thereby preventing transcription without inducing DNA cleavage [13]. CRISPR-Cas9 technologies have been established in a variety of bacterial genera, including *Bacillus*, *Escherichia*, *Clostridium*, and *Streptococcus* [14,15,16]. In LAB, a CRISPR/Cas9-mediated ssDNA recombineering platform has been applied to *Lactobacillus reuteri* and *L. lactis*, enabling gene deletions with efficiencies ranging from 8% to 50% [17,18]. Furthermore, a nisin-inducible, single-plasmid CRISPRi system has been reported for L. lactis, achieving more than a 50-fold reduction in upp transcription in the presence of the inducer [19].

Because CRISPRi relies on controlled expression of dCas9, precise regulation at the protein level is particularly critical. Leaky expression can obscure phenotypes or cause artificial toxicity, whereas insufficient induction may fail to uncover essential gene functions. This is especially relevant when studying essential genes or genes linked to toxic phenotypes, where even minor changes in basal activity can have significant consequences.

In this study, both plasmid-based and chromosomally integrated CRISPRi systems were constructed for *L. lactis*. The optimized chromosome-based platform was further employed to probe the function of the essential gene *ybeY*. Transcriptome analysis revealed that silencing *ybeY* strongly affected the expression of ribosomal proteins as well as numerous small RNAs, suggesting roles in ribosome homeostasis and RNA regulation.

## 2. Results

### 2.1. Establishment of an Inducible CRISPRi System with Fluorescently Tagged dCas9 in L. lactis

**Figure 1:**
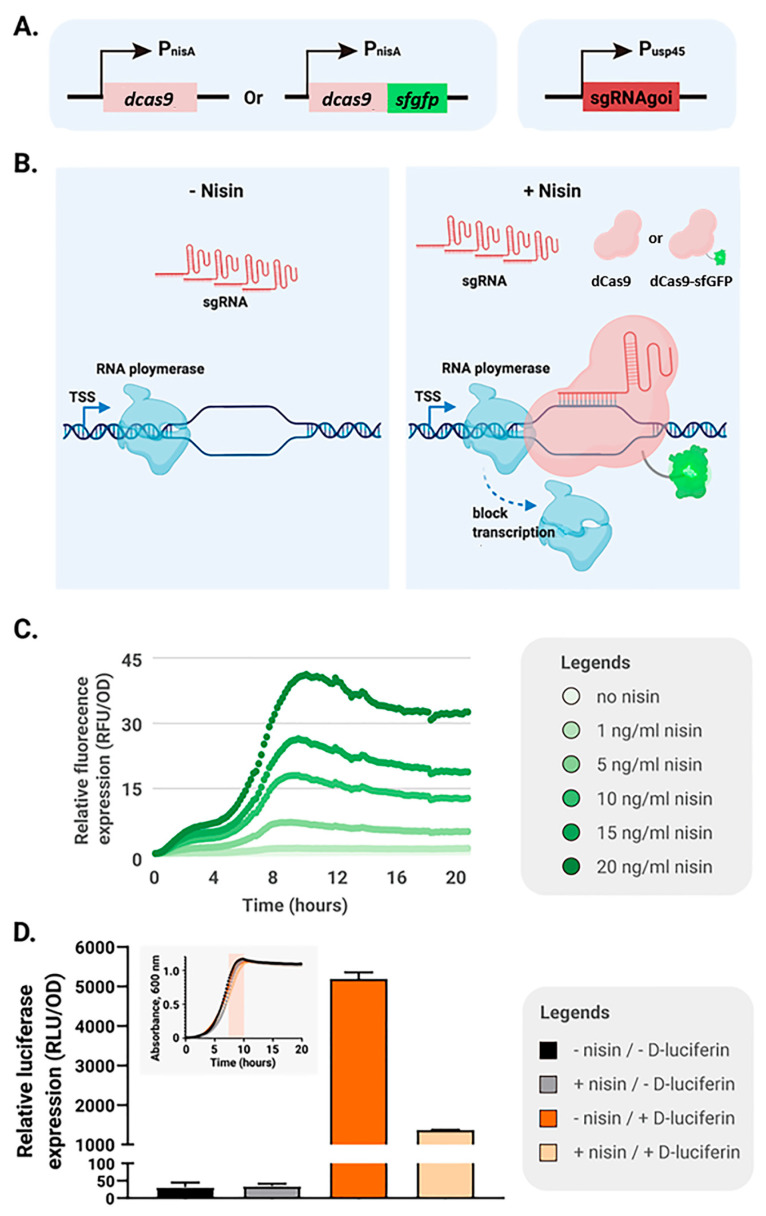
Nisin-inducible CRISPRi systems in *L. lactis*. (**A**) Two dCas9 expression modules were constructed: dCas9 alone or dCas9 fused with sfGFP (dCas9-sfGFP); each encoding gene was placed under the control of the nisin-inducible promoter P_nisA_. The sgRNA targeting a gene of interest (sgRNAgoi) was expressed from the constitutive P_usp45_ promoter. (**B**) Without nisin, sgRNA is produced but dCas9-sfGFP is absent, allowing transcription of the target gene. Upon nisin addition, both sgRNA and dCas9-sfGFP are expressed; the sgRNA guides dCas9-sfGFP to its target, and the resulting complex blocks transcription. The same applies to the construct with dCas9 alone. (**C**) Different levels of dCas9-sfGFP were induced in *L. lactis* NZ9000 (pNZ-P_nisA_-*dcas9-sfgfp*) by varying nisin concentrations. Cell density and fluorescence were monitored every 10 min. Values represent the mean of three replicates. (**D**) The CRISPRi system was validated in the luciferase reporter strain *L. lactis* NZ9000 *pseudo10*::P_usp45_-*luc*. Expression of dCas9-sfGFP was induced with 5 ng/mL nisin. OD_600_ and luciferase activity were measured every 10 min, and relative luciferase activity was calculated as luciferase/OD_600_. The maximum relative activity, observed at the end of exponential growth (highlighted by the orange bar in the inset growth curve), was used for the bar chart. Values are shown as the mean of triplicates.

To monitor expression levels, a fluorescent variant of dCas9 was generated by fusing its coding sequence at the 3′ end to the superfolder GFP (sfGFP) gene, connected through the flexible linker AGSGGEAEA [20,21]. For inducible expression, the dcas9 or dcas9-sfgfp gene was placed under the control of the nisin-responsive promoter P_nisA_ in a pNZ8048-derived plasmid [7], while the sgRNA cassette was constitutively expressed from the P_usp45_ promoter on a pTLR-based backbone (Figure 1A,B) [20,22]. sgRNA design followed previously established criteria to ensure efficient silencing: targeting of the non-template DNA strand, a minimum 12 bp base-pairing region, and complete sequence complementarity within the 7 nucleotides adjacent to the PAM site (5′-NGG-3′). To minimize off-target interactions, the 12 bp region immediately upstream of the PAM was selected [13]. The inducible host strain *L. lactis* NZ9000 was used [23]. To verify inducibility, fluorescence intensity was measured in NZ9000 harboring pNZ-P_nisA_-*dcas9*-*sfgfp*. Increasing concentrations of nisin (0–20 ng/mL) led to a dose-dependent rise in fluorescence, confirming controlled expression of dCas9-sfGFP (Figure 1C). Next, a luciferase reporter strain (NZ9000 *pseudo10*::P_usp45_-*luc*) was constructed to evaluate CRISPRi function. The chromosomal locus *pseudo10* is a commonly used silent region that allows stable chromosomal integration without affecting *L. lactis* growth. Cells carrying pNZ-P_nisA_-*dcas9*-*sfgfp* and pTLR-P_usp45_-sgRNA*luc* were analyzed for luciferase activity. Upon induction with 5 ng/mL nisin, luciferase activity decreased markedly, demonstrating that the dCas9-sfGFP fusion retained silencing activity (Figure 1D). At the end of exponential growth, a 74% reduction in luciferase expression was observed without detectable effects on bacterial growth.

### 2.2. Leaky dCas9 Expression Causes Basal Gene Repression

To further evaluate the functionality of the CRISPRi system, its effects on cell growth and morphology were examined. The gene *acmA*, which encodes the major autolysin responsible for cell separation and autolysis in *L. lactis* NZ9000 was selected as a target. Deletion of *acmA* results in the formation of extremely long chains of cells [24].

**Figure 2:**
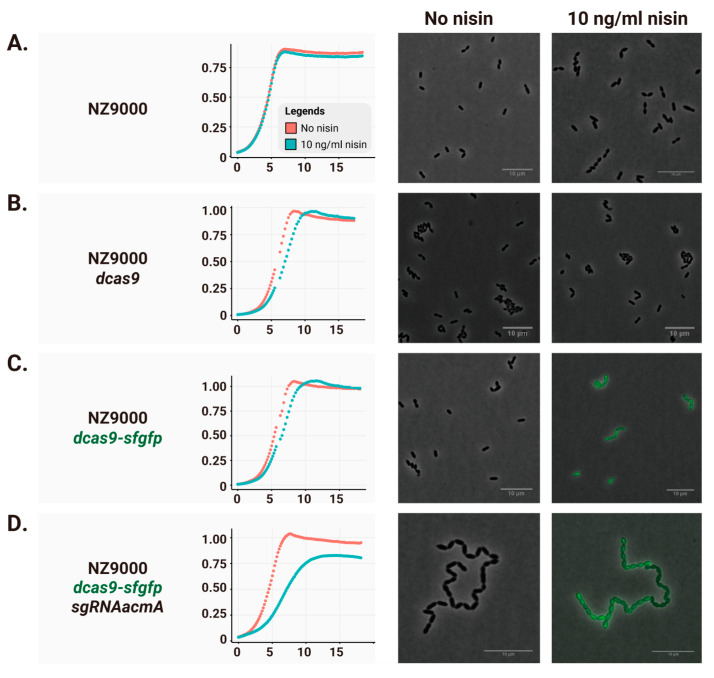
Plasmid-based CRISPRi-mediated silencing in *L. lactis*. Growth curves of strains cultured in CDM with (light blue) or without (red) 10 ng/mL nisin. Cell density was monitored every 10 min (mean of triplicates). Representative fluorescence images show corresponding morphological changes (scale bar = 10 μm). (**A**) NZ9000; (**B**) NZ9000 (pNZ-P_nisA_-*dcas9*); (**C**) NZ9000 (pNZ-P_nisA_-*dcas9*-*sfgfp*); (**D**) NZ9000 (pNZ-P_nisA_-*dcas9*-*sfgfp*; pTLR-P_usp45_-sgRNA*acmA*).

Adding nisin to strains expressing only dCas9 or dCas9-sfGFP had minimal impact on growth (Figure 2B,C, growth curves), whereas co-expression of the corresponding sgRNA targeting *acmA* caused a noticeable reduction in growth rate and final OD (Figure 2D). Microscopy analysis revealed that, as expected, long chains of cells were formed in the *acmA* knockdown strain (right micrographs in Figure 2A–D).

No detectable fluorescence from dCas9-sfGFP was observed in uninduced cells (left micrograph in Figure 2C). Nevertheless, long chains were present even without nisin induction (Figure 2D, left micrograph). These findings indicate that basal dCas9 expression resulting from P_nisA_ leakiness, while not affecting growth rate or final OD, is sufficient to block transcription and elicit the corresponding mutant phenotype.

### 2.3. Reducing Leaky Expression Through Chromosomal dCas9-sfGFP Insertion

**Figure 3:**
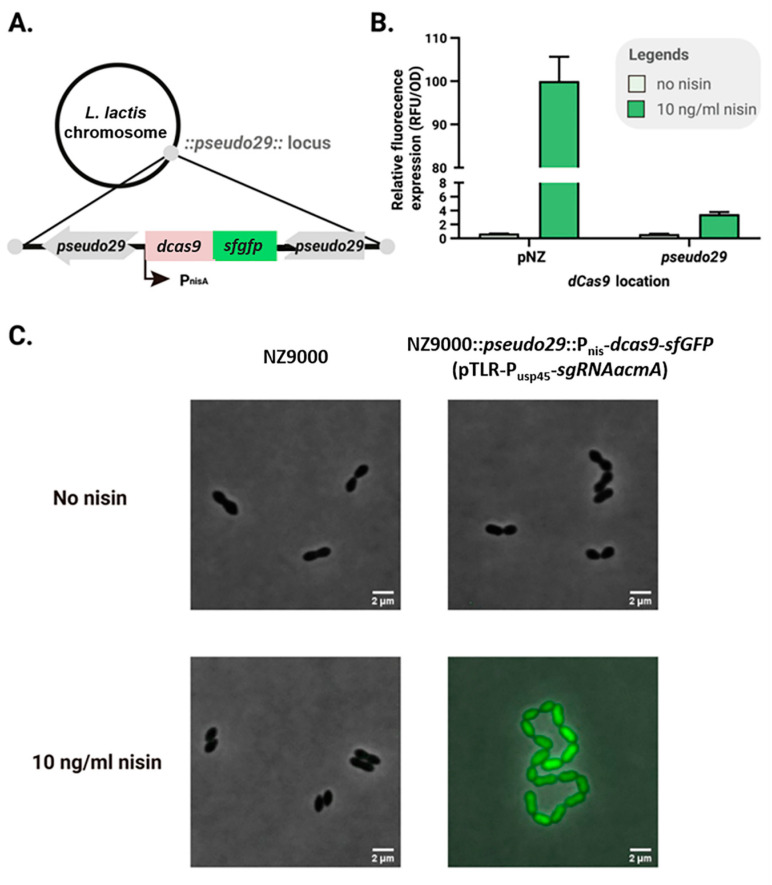
Chromosome-based CRISPRi provides tight control in *L. lactis*. (**A**) The P_nisA_-dcas9-sfgfp cassette was integrated into the *pseudo29* locus of NZ9000. (**B**) Fluorescence and OD_600_ were measured after 3 h of induction with or without 10 ng/mL nisin. Relative fluorescence was calculated as fluorescence/OD_600_ (mean of triplicates, error bars represent standard deviations, SD). (**C**) Representative fluorescence images of NZ9000 and the *acmA*-silenced strain CHML011 [NZ9000 *pseudo29*::P_nisA_-*dcas9*-*sfgfp* (pTLR-P_usp45_-sgRNA_*acmA*)]. Scale bar = 2 μm.

To minimize background activity of uninduced dCas9-sfGFP, the copy number of the dcas9-sfgfp cassette was reduced by chromosomal integration. The P_nisA_-dcas9-sfgfp construct was inserted into the transcriptionally silent *pseudo29* locus of *L. lactis* NZ9000 [25], while the sgRNA continued to be expressed constitutively from P_usp45_ on the plasmid pTLR. Expression levels of dCas9-sfGFP were compared between plasmid- and chromosome-based systems. Nisin (10 ng/mL) was added to cultures, and relative fluorescence was measured after 3 h of induction. As shown in Figure 3B, dCas9-sfGFP expression from the chromosome was approximately 20-fold lower than from the plasmid.

When targeting *acmA* with sgRNA in cells carrying the chromosomal dcas9-sfgfp cassette, strain NZ9000 *pseudo29*::P_nisA_-*dcas9*-*sfgfp* (pTLR-P_usp45_-sgRNA_*acmA*) displayed normal morphology in the absence of nisin. In contrast, the plasmid-based system showed observable phenotypic changes under the same conditions (compare Figure 3C with Figure 2D). These results demonstrate that chromosomal insertion of dCas9-sfGFP significantly reduces basal expression, allowing a tighter, more controllable chromosome-basedCRISPRi system, cbCRISPRi.

### 2.4. Application of Chromosomal-Based CRISPRi to Study ybeY Function

YbeY is a highly conserved protein found across nearly all sequenced bacterial species. It is typically involved in ribosome maturation and quality control as an endoribonuclease, and implicated in biofilm formation, stress responses, and virulence [26,27,28,29,30]. In *Sinorhizobium meliloti*, YbeY is required for sRNA-mediated silencing of amino acid ABC transporters [28], and in *Pseudomonas aeruginosa*, it regulates RpoS via the sRNA ReaL [27]. Its role in *L. lactis* remains unexplored, though it may function as a candidate sRNA chaperone in a species lacking Hfq, CsrA, or ProQ homologs [31,32,33,34,35].

Although *ybeY* is essential in several bacteria [36,37,38], it can be deleted in *P. aeruginosa*, *S. meliloti*, and *E. coli*, with pleiotropic effects on growth, toxin production, or cell aggregation [39,40]. Attempts to delete *ybeY* in *L. lactis* using the temperature-sensitive pGhost system or the pCS1966 oroP-based selection/counterselection system failed, even after multiple attempts (Figure S1) [9,41]. Standard methods relying on double-crossover homologous recombination failed to delete *ybeY* from the chromosome, despite multiple alternative attempts (Figure S1).

**Figure 4:**
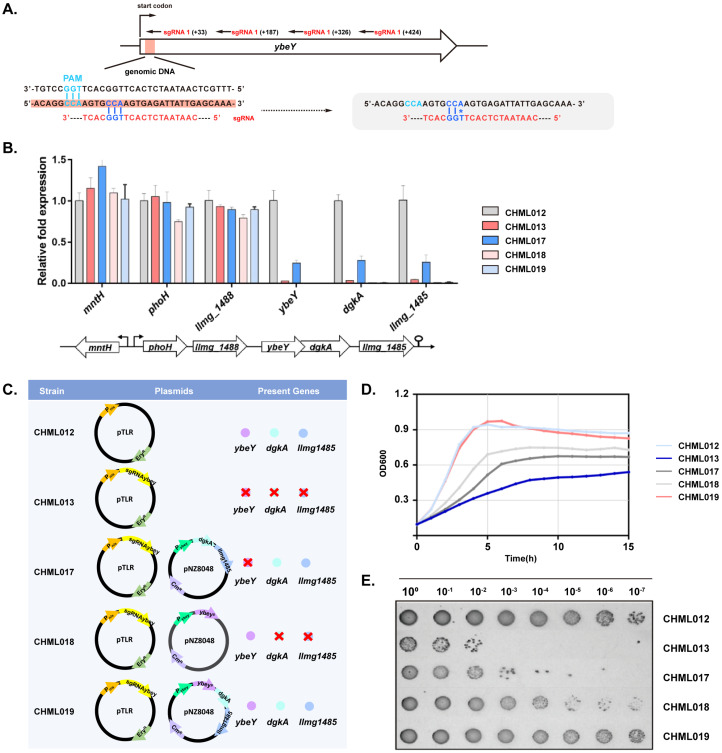
CRISPRi-mediated silencing of *L. lactis ybeY* and downstream genes. (**A**) Four sgRNAs (20 nt each) were designed to target different positions within *ybeY* (489 nt). Numbers in brackets indicate distance (nt) from the AUG start codon. The proline codon is highlighted (dark blue); its mutated version in *ybeYp* is marked with *. The red-shaded areas indicate the relative positions of the sgRNA target sites within the *ybeY* gene. (**B**) qPCR analysis of *ybeY* and five adjacent genes in the parental strain CHML012 [NZ9000 *pseudo29*::P_nisA_-dcas9] and four ybeY-silencing strains (CHML013–CHML016, carrying sgRNAybeY1–4) (mean of triplicates, error bars represent standard deviations, SD). All strains retained the intact chromosomal *ybeY* locus. (**C**) Plasmid constructs of the five strains, showing chromosomal targets silenced (red cross) and plasmid-based complementation. (**D**) Morphology of CHML012, the ybeY-silenced strain CHML013, and complementation strains CHML017–CHML019 carrying the indicated plasmids after induction with 10 ng/mL nisin. *ybeYp*: ybeY allele containing the proline codon mutation shown in (**A**). (**E**) Growth of plasmid-carrying strains on GM17 agar supplemented with 10 ng/mL nisin. qPCR and growth assays were performed twice with three replicates each.

Four sgRNAs targeting *ybeY* were designed for cbCRISPRi, and qPCR confirmed that all four (strains CHML013–CHML016) efficiently silenced transcription (Figure 4A,B). Three sgRNAs decreased *ybeY* expression by roughly 200-fold, while sgRNA2 exhibited weaker silencing. This lower efficiency is likely due to its 12-base region adjacent to the PAM having a GC content of about 33%, compared to 42–58% for the other sgRNAs. Silencing *ybeY* also reduced expression of downstream genes *dgkA*-*llmg1485* but did not affect upstream genes. Knockdown of the *ybeY*-*dgkA*-*llmg1485* operon caused severe growth defects (Figure 4D,E), while overexpression of *ybeY* in NZ9000 had no growth impact (Figure S2). To dissect the roles of the three genes in the observed growth defects, we constructed plasmid-based complementation strains. The first expressed YbeYp with a synonymous mutation at the sgRNA target site to prevent silencing (Figure 4A). The second complemented *dgkA* and *llmg_1485* under their native operon promoter, and the third co-expressed the mutated YbeYp together with *dgkA* and *llmg_1485*. As shown in Figure 4D,E, restoring only *ybeY* or *dgkA–llmg_1485* partially alleviated the growth defect, while co-expression of all three genes fully rescued it. These findings indicate that loss of YbeY has the strongest impact, but reduced expression of *dgkA* and/or *llmg_1485* also compromises growth.

**Figure 5:**
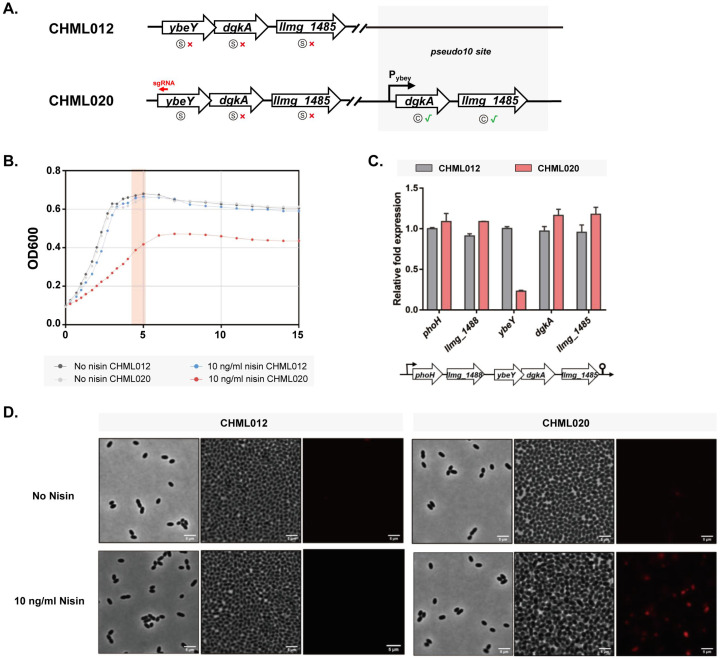
Growth and morphological effects of *ybeY* silencing in *L. lactis*. (**A**) Chromosomal organization of the analyzed strains. Red crosses indicate gene silencing, green check marks indicate complementation. (**B**) Growth of the parental strain CHML012 and the complemented strain CHML020. The red bar marks the time point of cell sampling for microscopy. (**C**) qPCR analysis of operon gene expression. (**D**) Representative microscopy images of stationary-phase cells with *ybeY* silenced (bottom row) or unsilenced (top row). Left and middle panels show low- and high-density regions of the same culture, respectively. Right panels display live/dead staining of the middle panel; red fluorescence indicates dead cells. Scale bar = 5 μm.

Plasmid-based complementation showed partial recovery when either *ybeY* or the two downstream genes were complemented, and complete recovery when all three were expressed (Figure 4D,E). To prevent overexpression, *dgkA*-*llmg1485* driven by their native promoter were integrated into the *pseudo10* locus (strain CHML20). This restored the growth defects while *ybeY* remained silenced (Figure 5B). No differences in the recovery of growth defects were observed between CHML020 and the plasmid-based *dgkA–llmg_1485* complementation strain CHML017. qPCR confirmed transcriptional restoration (Figure 5C). Light microscopy with live/dead staining indicated no viability difference in exponential phase, but stationary-phase cell death ranged from 15 to 54%, higher than 0.5–3% in the parent strain CHML012 (Figure 5D). These results confirm *ybeY* essentiality and the importance of downstream operon genes for normal growth of *L. lactis*.

### 2.5. Silencing of ybeY Alters Ribosomal and Other Gene Expression

Deletion of *ybeY* in several bacterial species results in the appearance of an additional band corresponding to 17S rRNA on agarose gels, representing the 16S rRNA precursor [29]. In *L. lactis*, however, knockdown of *ybeY* did not produce such a band (Figure S3). qPCR analysis also revealed no significant differences in the 5′ ends, middle regions, or 3′ ends of immature 16S rRNA and 23S rRNA (Figure S4). These findings suggest that *L. lactis* YbeY may not be primarily involved in 16S rRNA maturation, although a very low level of residual YbeY expression in the knockdown strain could suffice to complete this process.

**Figure 6:**
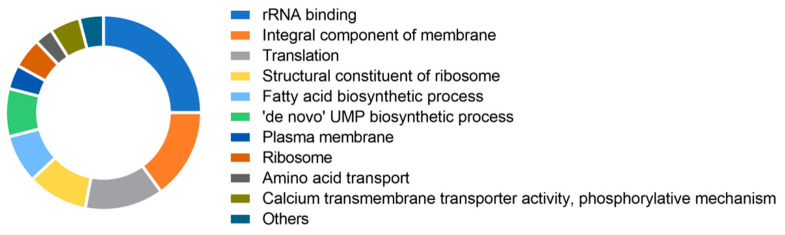
Transcriptomic impact of *ybeY* silencing in *L. lactis*. RNA-seq analysis of the *ybeY*-silenced strain at early stationary phase. Differentially expressed genes (DEGs) were subjected to functional annotation and gene set enrichment analysis to evaluate the global transcriptional response.

To further investigate *L. lactis* YbeY function, RNA-seq was performed comparing the transcriptomes of the *ybeY*-silenced strain and the control strain lacking the sgRNA-expressing plasmid, *L. lactis* NZ9000-dCas9. All strains were grown at 30 °C in GM17 medium to early log phase (OD600 = 0.4), followed by induction with 10 ng/mL nisin for 1 h to produce sgRNA and/or dCas9. Total RNA was isolated, rRNA-depleted, sequenced, and analyzed. This induction ensured sufficient dCas9 for function without causing severe side effects. RNA-seq revealed that *ybeY* silencing broadly affected the transcriptome, with most highly differentially expressed genes (DEGs) being downregulated. Functional and gene set enrichment analyses using FUNAGE-Pro [42] annotated DEGs to Gene Ontology (GO) terms (Figure 6). Many downregulated genes encode proteins of the 50S and 30S ribosomal subunits. Although no accumulation of immature 16S and 23S rRNA ends was detected, as was the case in *E. coli* and *P. aeruginosa* [27,29], ribosomal protein genes were largely repressed, indicating a role of YbeY in ribosome processing in *L. lactis*. Additionally, genes encoding membrane proteins, ATP synthase subunits, sugar/amino acid permeases, other transporters, and components of the *fab* and *acc* operons were downregulated. Some changes may reflect secondary effects after the 1 h induction.

The cbCRISPRi system was also applied to the 66-nt noncoding sRNA ArgX. Deletion of ArgX elevates *arc* and *arg* operon expression [43], and qPCR confirmed effective knockdown by CRISPRi with expected transcriptomic effects (Figure S5). Due to PAM constraints, only 42 of 184 sRNAs in *L. lactis* are potentially targetable (Table S2). Since YbeY has been reported to degrade an sRNA involved in oxidative stress in *P. aeruginosa* [27], sRNA expression in the *ybeY*-silenced strain was examined. Ten of 186 trans-encoded sRNAs and 2 of 60 *cis*-encoded antisense RNAs identified in *L. lactis* MG1363 [44] were differentially expressed. Whether YbeY directly or indirectly affects these sRNAs requires further study.

## 3. Discussion

The CRISPRi system provides a precise method for gene silencing in both microbial and higher organisms [45,46,47]. In bacteria, a functional CRISPRi platform requires two essential components: (i) an sgRNA that binds to the genome, with specificity determined by approximately 20 bp of base pairing with the target DNA and the protospacer adjacent motif (PAM), which in S. pyogenes is NGG; (ii) a dCas9 protein that forms a dCas9-sgRNA complex guided to its target, where it blocks transcription initiation or elongation depending on the sgRNA binding site. Each target gene requires an adapted 20 bp sgRNA sequence. Distributing the dcas9 and sgRNA genes on separate small plasmids is technically simpler than combining them on a single plasmid, which could exceed 10 kb, as the P_nisA_-*dcas9* cassette alone spans 3–5 kb, with or without *sfgfp*. Frequent replacement of the 20 bp sgRNA sequence on a single plasmid also risks backbone mutations, whereas separating the two cassettes reduces the likelihood of dCas9 mutations. Efficient cloning methods, such as quick-fusion cloning or golden-gate assembly, are preferred for rapidly changing sgRNA sequences; both strategies use a linearized sgRNA backbone, anneal commercial primers, and employ the product directly for subsequent cloning (Figure 1) [46,48].

These CRISPRi platforms are powerful tools for functional gene analysis in *L. lactis* and likely other LAB species. The cbCRISPRi system, in particular, provides a valuable addition to the genetic toolkit, enabling the study of essential genes, such as *ybeY*, which cannot be deleted in *L. lactis*. This platform allowed, for the first time, the investigation of *ybeY* function, despite challenges: incomplete depletion of YbeY and the substantial stress caused by its depletion, resulting in widespread transcriptomic effects. The absolute fluorescence values under uninduced conditions appear to be similar between the plasmid- and chromosome-based CRISPRi systems (Figure 3B). This is likely due to the detection limit of the plate reader, which may mask subtle differences at low fluorescence levels. Importantly, phenotypic assays (e.g., *acmA* silencing in Figure 2D) provide clear evidence that basal activity is reduced in the chromosomal system, confirming its functional advantage over the plasmid-based approach.

In this study, cbCRISPRi silencing of *ybeY* demonstrated that blocking transcript elongation with a single sgRNA can simultaneously suppress multiple genes within an operon. Complementation analysis is critical to confirm the contributions of downregulated genes. A synonymous codon mutation within the sgRNA target site can bypass CRISPRi; for instance, a single proline codon change fully counteracted sgRNA-mediated silencing of *ybeY*, whereas a single valine codon change partially restored growth (~70% of final OD). Generally, mutating two codons within the 12 bp core region adjacent to the PAM is recommended [13].

YbeY silencing in *L. lactis* did not reveal a clear role in 16S rRNA maturation, though residual protein expression from transcripts escaping silencing could suffice for this function. RNA-seq analysis suggests that YbeY may participate in rRNA maturation and/or ribosome quality control, consistent with homologous roles in other species. *E. coli* YbeY functions as an RNase that ensures 70S ribosome quality, working with RNase R to degrade rRNAs from defective 50S or 30S subunits [39]. In *E. coli* Δ*ybeY*, defective 70S ribosomes slightly accumulate, with a notable rise in free defective subunits [29]. Impaired translation due to these defective ribosomes results in growth defects and stress responses.

YbeY has also been implicated in sRNA regulation in several species [26,28,30,40]. In *E. coli*, 28 of 54 detectable sRNAs show YbeY-dependent expression changes [40]. Prior to this study, *L. lactis* YbeY had not been linked to sRNA regulation. Here, YbeY appears to influence certain sRNAs, with three upregulated candidates in the *ybeY* knockdown strain—LLMGnc_060, LLMGnc_129, and LLMGnc_172 (ArgX)—potentially being direct targets. Downregulation of other sRNAs suggests indirect effects or involvement of additional regulatory factors.

## 4. Materials and Methods

### 4.1. Bacterial Strains, Media, and Culture Conditions

The bacterial strains used in this study are listed in Table 1. *L. lactis* NZ9000 and its derivatives were grown at 30 °C in Difco M17 medium (BD, Franklin Lakes, NJ, USA) containing 0.5% (wt/vol) glucose (GM17). For fluorescence and luminescence analyses, *L. lactis* strains were grown in a chemically defined medium supplemented with 1% (wt/vol) glucose (CDM) [16]. When needed, erythromycin or chloramphenicol was added at a final concentration of 5 μg/mL. Chemically defined SA medium supplemented with 0.5% (wt/vol) glucose and 20 μg/mL 5-fluoroorotic acid (5-FOA; Sigma-Aldrich, St. Louis, MO, USA) as the sole pyrimidine source was used for the selection of chromosomal insertions [9]. *E. coli* DH5α was used as a cloning host; it was grown aerobically at 37 °C in LB media (Formedium, Norfolk, UK) supplemented with, when needed, erythromycin at a final concentration of 200 μg/mL. All chemicals were obtained from Sigma-Aldrich.

### 4.2. Recombinant DNA Techniques and Oligonucleotides

Standard molecular cloning was performed essentially as described previously [50]. Chromosomal DNA from *L. lactis* was isolated using the GenElute Genomic DNA Kit (Sigma-Aldrich). Plasmids and PCR products were isolated and purified using a High Pure plasmid isolation and PCR purification kit (Roche Applied Science, Mannheim, Germany) and a NucleoSpin gel and PCR cleanup kit (Macherey-Nagel, Düren, Germany) according to the manufacturers’ instructions. PCR was performed with Phusion or DreamTaq polymerase (both from Fermentas, St. Leon Roth, Germany) according to the manufacturer’s protocol. The obtained PCR products were mixed and treated with a mixture of Quick-Fusion enzymes (BIO-Connect Services BV, Huissen, The Netherlands), yielding 15-nucleotide overhangs that were annealed to complementary overhangs. No ligation was needed; Quick-Fusion-treated mixtures were directly used to transform *E. coli*. The oligonucleotides used in this study are listed in Supplementary Table S1 and were purchased from Biolegio BV (Nijmegen, The Netherlands). Competent *E. coli* cells were transformed using heat shock [51], while electrocompetent *L. lactis* cells were transformed using electroporation [52] with a Bio-Rad gene pulser (Bio-Rad Laboratories, Richmond, CA, USA). All nucleotide sequencing was performed at Macrogen Europe (Amsterdam, The Netherlands).

### 4.3. Construction of Plasmid-Based CRISPRi Systems

Pertinent regions of all the plasmids constructed in this study were sequenced to confirm their proper nucleotide sequences. *Streptococcus pyogenes dcas9* (*dcas9*) was obtained from plasmid pJWV102 [46] by PCR using the primers 0032-USER_Pnis_dCas9_F and 0220-dCas9-USER_R, while linearized pNZ8048 vector was obtained using the primers 0217-pNZ8048_USER_F/0221-Pnis_pNZ8048_R. The fragments were fused employing the USER cloning kit (New England Biolabs, Ipswich, MA, USA) according to the manufacturer’s instructions, with the exception of using only one half of the recommended volume per reaction. The reaction mixture was directly used to transform competent *L. lactis* NZ9000. The resulting vector was labeled pNZ-P_nisA_-*dcas9*. The plasmid pNZ-P_nisA_-*dcas9-sfgfp*, which expresses a Cas9 variant that couples C-terminally with sfGFP via the flexible linker sequence AGSGGEAEA, was generated as follows: *dcas9* was amplified from pJWV102 using the primers 0032-USER_Pnis_dCas9_F/0218-dCas9_USER_R. The *sfgfp* gene with the polylinker-encoding sequence at its 5′ end was amplified from the plasmid pLG-MG1 [21] using the primers 0215-sfGFP_USER_F/0219-linker_sfGFP_USER_R. The fragments were fused through USER cloning, and the mixture was subsequently used to transform competent *L. lactis* NZ9000 to obtain the proper construct.

The DNA fragment encoding the single guide RNA targeting the luciferase gene (*sgRNAluc*) was amplified from the plasmid pPEPX-P3-*sgRNAluc* [46] by the primers 0149-sgRNA_F/0150-sgRNA_R. The constitutive *L. lactis* promoter P_usp45_ was amplified from the plasmid pSEUDO::Pusp45-*sfgfp(Bs)* [20] with the primers 0147-Pusp45_F/0148-Pusp45_R, and the linearized pGHost vector was obtained after amplification using the primers 0145-pGHost_F/0146-pGHost_R. The three fragments were then fused with the USER cloning kit, and the reaction mixture was subsequently used to transform competent *E. coli* DH5α, resulting in the plasmid pGHost-P_usp45_-*sgRNAluc*. The P_usp45_-*sgRNAluc* cassette, obtained by double digestion of the latter plasmid with the restriction enzymes NcoI and XhoI, was ligated into pTLR [22] treated with the same enzymes. The ligation mixture was used to transform competent *E. coli* DH5α, yielding the plasmid pTLR-P_usp45_-*sgRNAluc*. The *sgRNAluc* sequence is transcribed directly after +1 of the P_usp45_ promoter and contains 20 nucleotides (nt) as the base-pairing region, which targets the luciferase gene and is followed by an optimized sgRNA [53]. The plasmid pTLR-P_usp45_-*sgRNAluc* was used as the template for the generation of other sgRNA expression plasmids by quick-fusion cloning (see the next section).

### 4.4. Quick-Fusion Cloning of sgRNA Genes

The 20-nt guide sequences of sgRNAs targeting different genes were selected with the CRISPR Primer Designer [54]. Briefly, a search was performed within the coding sequence of each gene for a 14-nt specificity region consisting of the 12-nt “seed” region of the sgRNA and GG of the 3-nt PAM (GGN). sgRNAs with more than one binding site within the *L. lactis* genome, as determined by BLAST (v 2.12.0) searches, were discarded. The guide sequence was chosen to be as close as possible to the 5′ end of the coding sequence of the targeted gene [13]. Cloning of sgRNA sequences was performed by quick-fusion cloning (Figure S6). The primer pair 0153-sgRNA_backbone_FW/0154-sgRNA_backbone_RV was designed for linear amplification of the plasmid pTLR-P_usp45_-*sgRNAluc*. The primers bind directly upstream and downstream of the 20 bp guide sequence to enable easy sgRNA sequence swapping. To fuse the new 20-nt guide sequence into the linearized vector, two 50-nt complementary primers were designed for each target gene. Each primer contained 15 nt at one end, overlapping with the sequence on the 5′ end of the linearized vector, followed by the 20-nt gene-specific guide sequence and then 15 nt overlapping with the sequence on the 3′ end of the linearized vector. The two 50-nt complementary primers were annealed in TEN buffer (10 mM Tris, 1 mM EDTA, 100 mM NaCl, pH 8) by heating at 95 °C for 5 min and slowly cooling to room temperature. The annealed product was fused with the linearized vector using the Quick-Fusion Cloning Kit (BIO-Connect Services BV) according to the manufacturer’s instructions, except for using only one half of the recommended volume per reaction. The reaction mixture was directly used to transform competent *E. coli* DH5α, and the designated plasmid was subsequently introduced into the strains CHML009 (NZ9000 *pseudo29*::P_nisA_-*dcas9*) or CHML010 (NZ9000 *pseudo29*::P_nisA_-*dcas9-sfgfp*).

### 4.5. Construction of the Luc Reporter Strain

The *luc* gene was amplified from the plasmid pPEP23 [55] using the primers 0189_luc_F/0190-luc_R, while the vector pSEUDO::Pusp45-*sfgfp(Bs)* [20] was linearized by amplification using the primers 0187-pseudo10_R/0188-pseudo10_F. The two fragments were fused by employing the Quick-Fusion Cloning Kit protocol. The reaction mixture was directly used to transform competent *E. coli* DH5α to pick up plasmid pSEUDO10-P_usp45_-*luc*. This plasmid, which cannot replicate in *L. lactis*, was introduced into *L. lactis* NZ9000 via electroporation. Cells in which the plasmid had integrated into the genomic locus *pseudo10* were obtained by selection on erythromycin. The proper integrant strain, NZ9000::*pseudo10*::P_usp45_-*luc*, was subsequently selected by growing on selective SA media plates containing 20 μg/mL 5-FOA [9].

### 4.6. Construction of a Genome-Based CRISPRi System

The flanking regions of *pseudo29* were amplified using 0247-pseudo29_UP_F/0248-pseudo29_UP_R and 0249-pseudo29_DOWN_F/0250-pseudo29_DOWN_R, while the linearized vector pCS1966 was obtained by PCR amplification using the primer pair pCS1966_1FW/pCS1966_1RV. The fragments were fused by Quick-Fusion Cloning as described above, after which the reaction mixture was used to transform competent *E. coli* DH5α. The resulting vector was designated pSEUDO29. The P_nisA_-*dcas9-sfgfp* cassette was amplified using the primers 03-dcas9-pseudo29-F/04-dcas9-pseudo29-R, while linearized pSEUDO29 was obtained by amplification employing the primers 01-pseudo29_F/02-pseudo29-R. The fragments were fused with a Quick-Fusion Cloning Kit. A transformant carrying pSEUDO29-P_nisA_-*dcas9-sfgfp* was selected in *E. coli* DH5α. This plasmid, which does not replicate in *L. lactis*, was introduced into *L. lactis* NZ9000 via electroporation; cells in which a two-step homologous recombination event occurred were selected on selective SA medium plates supplemented with 20 μg/mL 5-FOA [9]. The obtained strain was labeled NZ9000::*pseudo29*::P_nisA_-*dcas9-sfgfp*.

### 4.7. Optical Density, Fluorescence, and Luminescence Measurements

*L. lactis* was grown overnight in CDM at 30 °C and then diluted to an optical density (OD) at 600 nm (OD_600_) of 0.1 in fresh CDM. The mixture was incubated until the mid-log phase (OD_600_ = 0.5) was reached, after which the cells were diluted again to a starting OD_600_ of 0.05 in fresh CDM supplemented with different concentrations of nisin. Then, each culture was dispensed to 200 μL, in triplicates, in wells of a 96-well microtiter plate (Polystyrol, transparent, flat, and clear bottom; Corning, New York, NY, USA). The OD_600_ and fluorescence signal (GFP: excitation 485 nm/emission 535 nm) were measured every 10 min at 30 °C in an Infinite 200 Pro plate reader with I-control 1.10.4.0 software (Tecan Group Ltd., Männedorf, Switzerland). For luminescence measurements, the cells were grown as above, except that when the cells were diluted to OD_600_ = 0.05, D-luciferin sodium salt (SYNCHEM OHG, Altenburg, Germany) was added to a final concentration of 2.5 mg/mL. The cultures were subsequently divided in triplicate (200 μL each sample) in 96-well polystyrol microtiter plates (white, flat, and clear bottom; Corning). The OD_600_ and luminescence signals were measured every 10 min at 30 °C in an Infinite 200 Pro plate reader with I-control 1.10.4.0 software.

### 4.8. Microscopy

All micrographs were obtained with a DeltaVision Elite inverted epifluorescence microscope (Applied Precision, GE Healthcare, Issaquah, WA, USA) equipped with a stage holder, a climate chamber, a seven-color combined set InsightSSI solid-state illumination module, and a scientific complementary metal oxide semiconductor (sCMOS) camera (PCO AG, Kelheim, Germany). A 100× phase-contrast objective (numerical aperture 1.4, oil immersion, DV) was used for image capture in combination with SoftWorX 3.6.0 software (Applied Precision, GE Healthcare) to control the microscope setup. A fluorescence filter set with excitation at 475/28 nm and emission at 525/48 nm was used to visualize GFP. Fluorescence was imaged with 0.5 to 1 sec exposure times, while the maximum transmission of the light source was maintained for all exposure settings. A standard microscope slide was prepared with a layer of solidified agarose (1.5%, wt/vol in phosphate-buffered saline), and 1 μL of a bacterial cell culture was spotted onto the agar. The sample was covered with a standard microscope coverslip for microscopic observation.

### 4.9. Cell Viability Test

Cells were cultured overnight at 30 °C in Difco GM17 medium, transferred to fresh medium to an OD_600_ of 0.05 and grown to the early log phase (OD_600_ = 0.4). dCas9 protein expression was induced for 3 h with 10 ng/mL nisin, after which the cells were 10-fold serially diluted. Four microliters of solution were dropped on GM17 agar supplemented with the proper antibiotic, and the mixture was incubated at 30 °C for 24 h for observation.

### 4.10. RNA Isolation and Quality Control

All procedures were executed at 4 °C, and all solutions were treated with DEPC and subsequently sterilized. The cells were grown to the required OD_600_ via induction, if necessary, and pelleted by centrifugation. The cells were rapidly frozen in liquid nitrogen. For RNA isolation, the samples were resuspended in 400 μL of TE buffer (10 mM Tris-HCL, 1 mM EDTA, pH 7.4). SDS (to 1%), 0.5 g of glass beads (75–150 µm; Thermo Fischer Scientific, Rockford, IL, USA) and 500 μL of phenol/chloroform (1:1 *v*/*v*) were added, after which the cells were disrupted by shaking 2 times for 60 s, with a 1 min interval on ice, in a Biospec Mini-BeadBeater (Biospec Products, Bartlesville, OK, USA). The cell suspension was centrifuged at 14,000 rpm for 15 min, and the upper liquid containing nucleic acids was removed and treated with 250 μL of chloroform. The nucleic acids in the water phase were obtained after centrifugation at 400 μL and were precipitated by adding sodium acetate to 0.3 M and 2.5 volumes of 100% ethanol and incubating overnight at −80 °C. The nucleic acid pellet was resuspended in 100 μL DNase digestion buffer with 85 μL DEPC-Milli-Q water, 10 μL 10× DNase I buffer, and 5 μL DNase I (Roche Diagnostics GmbH, Mannheim, Germany) and incubated for 1 h at 37 °C. RNA was then purified using standard phenol/chloroform extraction and sodium acetate/ethanol precipitation. The RNA pellets were resuspended in 100 μL of DEPC-treated MiliQ water. The RNA concentration was measured with a Nanodrop ND-1000 (Thermo Fisher Scientific, Waltham, MA, USA). The integrity of the 16S/23S rRNA and the presence of any DNA contamination were assessed by both gel electrophoresis and analysis on an Agilent 2100 Bioanalyzer (Agilent Technologies, Waldbronn, Germany). The RNA samples were stored at −80 °C.

### 4.11. Real-Time PCR (RT-qPCR)

cDNAs were synthesized using the SuperScript™ III One-Step RT-PCR System and random hexamer primers (Thermo Fisher Scientific) in 20 μL reaction volumes. RT-PCR was performed using SYBR II Green Supermix and an iCycler Thermal Cycler with an iQ5 Multicolor Real-Time PCR Detection System (all three from Bio-Rad Laboratories). All the samples were analyzed in triplicate. Since *ybeY* might be involved in 16S maturation in *L. lactis*, the *gyrA* gene was used as an internal control instead of the 16S gene [56]. All the experiments were performed twice with 3 replicates.

### 4.12. RNA Sequencing, Data Analysis and Visualization

All RNA preparations for sequencing were made from 2 biological replicates. rRNA depletion and cDNA library preparation were performed using RiboCop rRNA Depletion and total RNA-Seq Library Prep Kits (Lexogen GmbH, Wien, Austria). cDNA libraries were sequenced using Illumina Next-Generation Sequencing technology. The raw sequencing reads were mapped to the *L. lactis* MG1363 reference genome (accession: NC_009004.1). Read alignment was performed on Bowtie [57]. The reads per kilobase of transcript and per million mapped reads (RPKM) values were used as inputs for the T-REx2 analysis pipeline [58] and the FUNctional Analysis and Gene Set Enrichment for Prokaryotes online tool (FUNAGE-Pro) [42] for global statistical analyses. As a large number of genes were widely affected, DEGs were visualized according to the following criterion: >3-fold change in expression (|Log2FC| ≥ 1.5) was considered to indicate statistical significance. The top hit genes were weighted-selected from gene set enrichment analysis. sRNAs with a 1.5-fold change in expression and statistical significance were selected (*p*-value < 0.01).

## Figures and Tables

**Table 1:** Strains used in this study.

Strains	Relevant Genotype and Plasmids	Source
DH5α	DH5α	[49]
NZ9000	MG1363, *pepN::nisRK*	[23]
CHML001	NZ9000 (pNZ-P_nisA_-*dcas9*)	This study
CHML003	NZ9000 (pNZ-P_nisA_-*dcas9-sfgfp*)	This study
CHML004	NZ9000 (pNZ-P_nisA_-*dcas9-sfgfp* & pTLR-P_usp45_-*sgRN*A*luc*)	This study
CHML005	NZ9000 (pNZ-P_nisA_-*dcas9-sfgfp* & pTLR-P_usp45_-*sgRNAacmA*)	This study
CHML007	NZ9000 *pseudo10*::P_usp45_-*luc*	This study
CHML008	NZ9000 *pseudo10*::P_usp45_-*luc* (pNZ-P_nisA_-*dcas9-sfgfp* & pTLR-P_usp45_-*sgRNAluc*)	This study
CHML009	NZ9000 *pseudo29*::P_nisA_-*dcas9*	This study
CHML010	NZ9000 *pseudo29*::P_nisA_-*dcas9-sfgfp*	This study
CHML011	NZ9000 *pseudo29*::P_nisA_-*dcas9-sfgfp* (pTLR-P_usp45_-*sgRNAacmA*)	This study
CHML012	NZ9000 *pseudo29*::P_nisA_-*dcas9* (pTLR)	This study
CHML013	NZ9000 *pseudo29*::P_nisA_-*dcas9* (pTLR-P_usp45_-*sgRNAybey*)	This study
CHML014	NZ9000 *pseudo29*::P_nisA_-*dcas9* (pTLR-P_usp45_-*sgRNAybey2*)	This study
CHML015	NZ9000 *pseudo29*::P_nisA_-*dcas9* (pTLR-P_usp45_-*sgRNAybey3*)	This study
CHML016	NZ9000 *pseudo29*::P_nisA_-*dcas9*(pTLR-P_usp45_-*sgRNAybey4*)	This study
CHML017	NZ9000 *pseudo29*::P_nisA_-*dcas9* (pTLR-P_usp45_-*sgRNAybey &* pNZ-P_ybey_*-dgkA-llmg1485*)	This study
CHML018	NZ9000 *pseudo29*::P_nisA_-*dcas9* (pTLR-P_usp45_-*sgRNAybey &* pNZ-P_ybey_*-ybey^p^*)	This study
CHML019	NZ9000 *pseudo29*::P_nisA_-*dcas9* (pTLR-P_usp45_-*sgRNAybey &* pNZ-P_ybey_*- ybey^p^-dgkA-llmg1485*)	This study
CHML020	NZ9000 *pseudo29*::P_nisA_-*dcas9, pseudo10::*P_ybey_*-dgkA-llmg1485* (pTLR & pNZ-P_usp45_-*sgRNAybey*)	This study

## Data Availability

The data presented in this study are available on request from the corresponding author due to reasonable request.

## Supplementary Materials

The following supporting information can be downloaded at: https://www.mdpi.com/article/10.3390/ijms26199516/s1.

## Abbreviations

The following abbreviations are used in this manuscript:

LAB	lactic acid bacteria
NICE	nisin-controlled expression
CRISPR-Cas	clustered regularly interspaced short palindromic repeats and CRISPR-associated proteins
DSB	double-strand break
crRNA	CRISPR RNA
tracrRNA	trans-activating RNA
dCas9	catalytically inactive variant of Cas9
CRISPRi	CRISPR interference
sfGFP	superfolder GFP
DEGs	differentially expressed genes
GO	Gene Ontology
