# Supplementary material for: Chromosomal and Plasmid-Based CRISPRi Platforms for Conditional Gene Silencing in Lactococcus lactis
Source: Int J Mol Sci. 2025 Sep 29;26(19):9516. doi: 10.3390/ijms26199516 (PMC12524933; doi:10.3390/ijms26199516)
Supplement: Supplementary file 1 [file ijms-26-09516-s001.zip › ijms-3851844-supplementary.pdf]

SUPPLEMENTAL MATERIALS

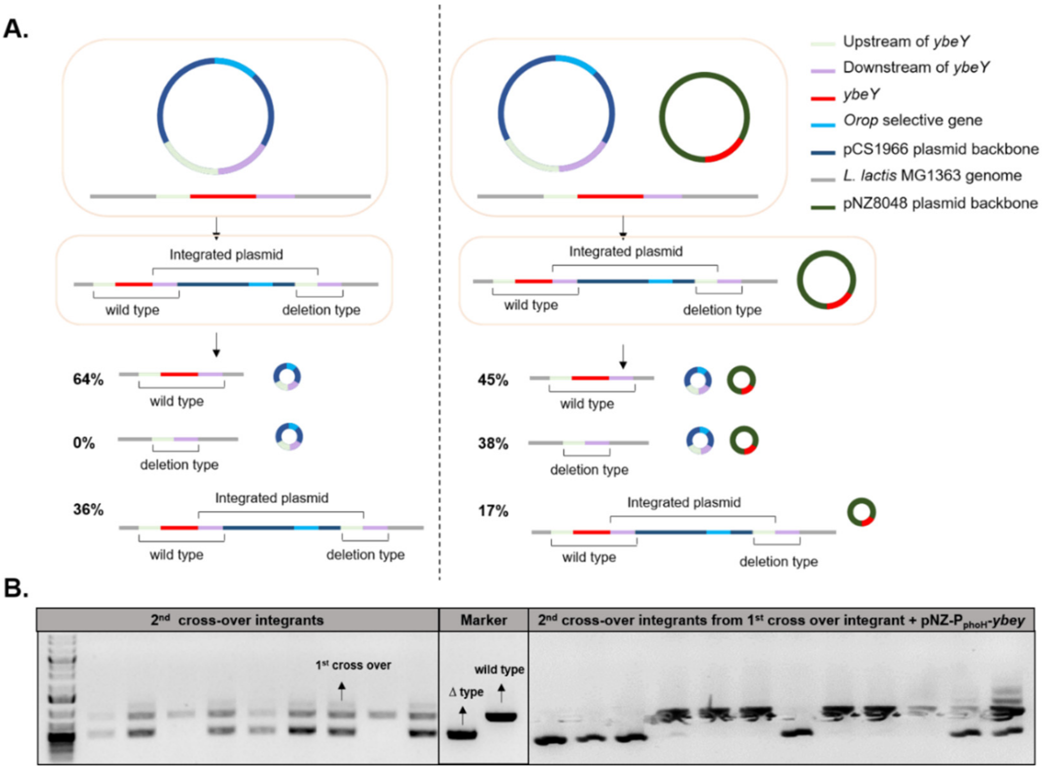

Figure S1. The genomic information and expression of the essential gene *ybeY* in *L. lactis*. (A) Deletion of *ybeY* can only be achieved in the presence of a plasmid carrying *ybeY*. (B) Agarose gel showing PCR results of the genetic types (wild type or *ybeY* deletion (Δ type)) of 2nd crossover integrant strains. Without the providing *ybeY* on a complementation plasmid, chromosomal knockout of *ybeY* cannot be achieved. However, when *ybeY* expression was driven by its original promoter, on plasmid pNZ-P$_{phoA}$-*ybeY*, a knockout mutant can be easily generated. For the 2nd crossover integrants in the left panel, more than 300 colonies were checked, with 4 independent experiments, each one slightly modifying the conditions to improve crossover efficiency. For the 2nd crossover integrants from the 1st crossover integrants, with the plasmid expressing *ybey* (right panle), 31 colonies were checked. The *ybeYn* deletion (genetic Δ type) was readily obtained.

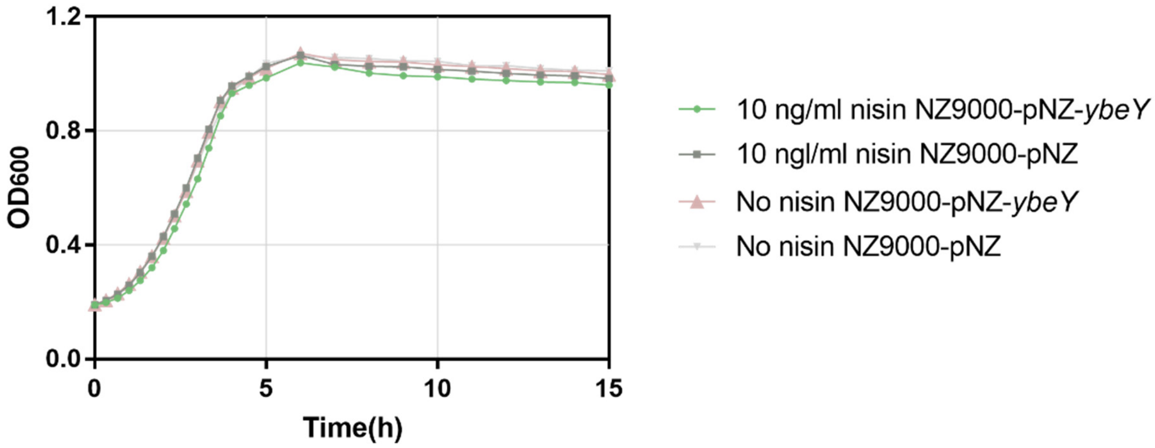

Figure S2 Growth curves of *ybeY*-overexpressing strain. Nisin-induced overexpression of *ybeY* does not result in growth differenceswhen compared with the uninduced strain.

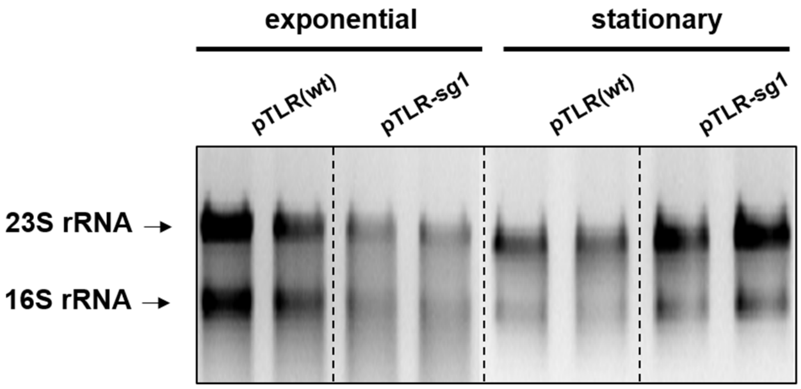

Figure S3 Total RNA isolated from strains carrying the indicated plasmids. Two clear bands corresponding to 23S and 16S rRNA are visible in all cases. No band of the 16S precursor 17S rRNA appeared, unlike what has been reported in some bacterial species, such as *P. aeruginosa* [27].

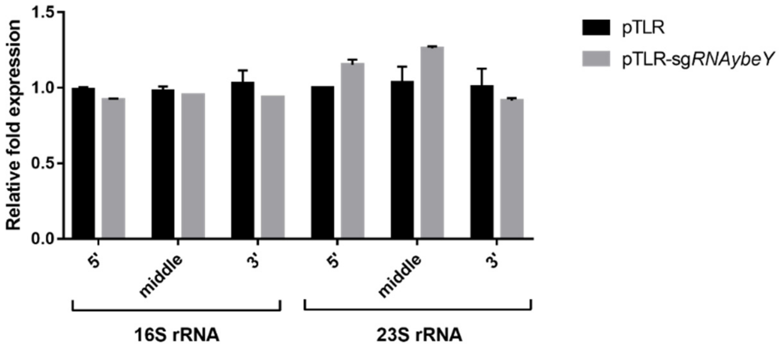

Figure S4. RT–qPCR of the 5′-end, middle and 3′-end of 16S and 23S rRNA.

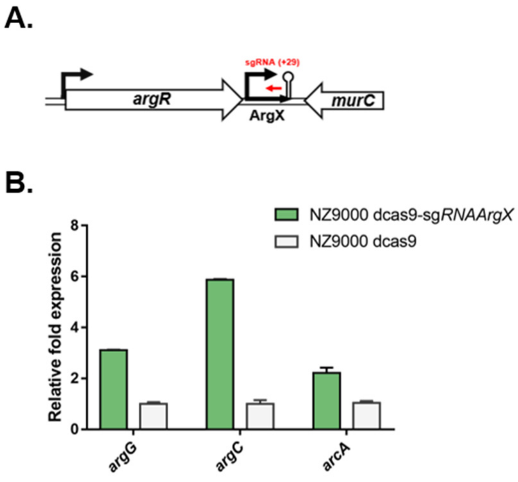

Figure S5. Silencing of the sRNA ArgX. (A) The sRNA ArgX (solid black arrow) is transcribed from the 3′-end of the *argR* gene. The sgRNA was designed to target the middle of the *argX* gene. (B) qPCR of the *arc* and *arg* operons. Knockdown of *argX* led to the upregulation of the gene in the operons. This trend was the same as that observed for the de ArgX deletion mutant.

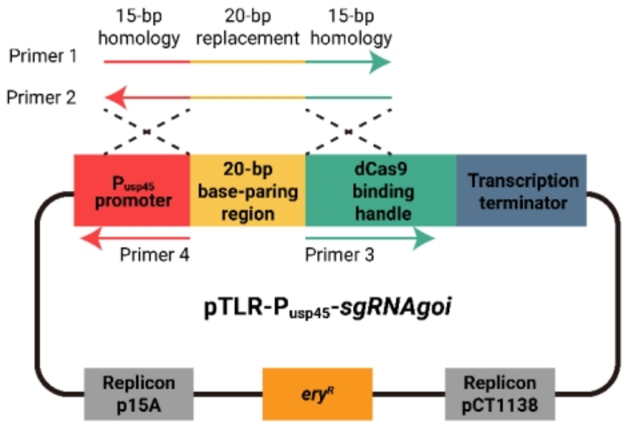

Figure S6. Quick-fusion cloning strategy for sgRNA construction. Schematic overview of the quick-fusion method used to generate sgRNA expression plasmids. For each target gene, two complementary primers (primers 1 and 2) were designed, each containing a 15-bp homology stretch flanking the 20-bp sgRNA targeting sequence. The pTLR vector was linearized by PCR using primers 3 and 4, enabling repeated replacement of the 20-bp region. Replication origins p15A and pCT1138 support plasmid maintenance in E. coli and L. lactis, respectively. eryR, erythromycin resistance marker.

Table S1 Sequences of oligonucleotides used for plasmid and strain construction

Primer name	Sequence (5’ –> 3’)
0220-dCas9-USER_R	ACTTATAAAGCUCTCGAGGTCGACTTAGTCAC
0217-pNZ8048_USER_F	AGCTTTATAAGUAATTACAGCACGTGTTGCTTTGATTG
0221-Pnis_pNZ8048_R	AGTATAATATGTTTAGCTGGTTTATAAAAAGCGAGGTTTAAGAGCTATGC
0218-dCas9_USER_R	AGTCACCTCCUAGCTGACTCA
0215-sfGFP_USER_F	AGGAGGTGACUAATGTCAAAAGGAGAAGAGCTGTTC
0219-linker_sfGFP_USER_R	AGGAGGTGACUTGCAGGCGGGATCTGGTG
0149-sgRNA_F	AGAGGATAGAAUGGCGCCGT
0150-sgRNA_R	AGACTTATTTACUGTTACTGGAGGGATCCATGAG
0147-Pusp45_F	AATATGAACATGAUTATATTTACTAATCGCTGGACA
0148-Pusp45_R	ATTCTATCCTCUTAAACATATTATACTATTCCTACCCCA
0145-pGHost_F	ATCATGTTCATATUTATCAGAGCTC
0146-pGHost_R	AGTAAATAAGTCUAGTGTGTTAGAC
0189_luc_F	ATAAGGAGGACAAACATGAGATCCGCCAAAAACATAAAGAAAG
0190-luc_R	ACTAGTGCTCATTATTTATTATTACAATTTGGGCTTTCCGC
0221_sgRNA(acmA)_F	AGTATAATATGTTTAGCTGGTTTATAAAAAGCGAGGTTTAAGAGCTATGC
0222_sgRNA(acmA)_R	GCATAGCTCTTAAACCTCGCTTTTTATAAACCAGCTAAACATATTATACT
0235-sgRNA(ftsZ)	AGTATAATATGTTTATTCTTCAATCATACGGTTGAGTTTAAGAGCTATGC
0236-sgRNA(ftsZ)	GCATAGCTCTTAAACTCAACCGTATGATTGAAGAATAAACATATTATACT
0237-sgRNA(pbp2B)	AGTATAATATGTTTAGATAATCTGAACATCGCCACGTTTAAGAGCTATGC
0238-sgRNA(pbp2B)	GCATAGCTCTTAAACGTGGCGATGTTCAGATTATCTAAACATATTATACT
0187-pseudo10_R	GTTTGTCCTCCTTATTAGTTAATCAGTA
0188-pseudo10_F	ATAATGAGCACTAGTCAAGGTCGG
0247-pseudo29_UP_F	CGCTCACAATTCCACAGCAGAAATGTATGTGGTCAGC
0248-pseudo29_UP_R	TCTTATTCGCGCAAATTAGTCACATTAGCCGAGTGACACCTT
0249-pseudo29_DOWN_F	GTGTCACTCGGCTAATGTGACTAATTTGCGCGAATAAGATGA
0250-pseudo29_DOWN_R	TCACTCATTAGGCACTCAGATAAACGCATTTTCGCTCT
pCS1966_1FW	GTGCCTAATGAGTGAGCTAACTC

Primer name	Sequence (5' –> 3')
pCS1966_1RV	GTGGAATTGTGAGCGGATAAC
03-dcas9-pseudo29-F	GGTTAAAGGTGTCACTCGGCCTAATGTCACTAACCTGCCCCGTT
04-dcas9-pseudo29-R	TCAGCCTCATCTTATTCGCGCGCTTTGATTGTTCTATCGAAAGCG
01-pseudo29_F	GCGCGAATAAGATGAGGCTGA
02-pseudo29-R	GCCGAGTGACACCTTTAACC
0121-Pusp45_seq_F	AGAGAGGAAGAAGAAGCATAGGA
0123-GFP_seq_R	GCTATTGTTTCTGGAGCCAATTG
0158-pGHost_seq_F	GCTGCAAGGCGATTAAGTTG
0159-pGHost_seq_R	GACAACATCTTCGCTGCAAAG
0168-pTLR_seq_F	CTATATCGTTAGGTACAGCTTCC
0170-pTLR_seq_R_2	CGTGGCCAATATGGACAACTT
0182-dCas_Seq_6	TCGTCTTGATTTACTCAATCGTGC
0227-Seq_luc_1	TATCGGAGTTGCAGTTGCGC
0228-Seq_luc_2	GATGATAAACCGGGCGCGGT
0263-pNZ_seq_FW	ACAGTCGGTTTTCTAATGTCACT
0264-pNZ_seq_RV	CAGCAATATCAGTAATTGCTTTATC
0332-pseudo10-seq_F	CTGGAGCCAATTGGCATTGGA
0333-pseudo10-seq_R	GAGGACAAAAGTAATCATTTGACTATTG
0020_dCas9 _seq_R	GCTTCTTGTCTTCTTCCACCA
0021_dCas9 _seq_F_1	GGTAGATTCTACTGATAAAGCGGAT
0022_dCas9 _seq_F_2	GCAAGCAACGGACCTTTGACA
0023_dCas9 _seq_F_3	GCTGATCCATGATGATAGTTTGA
0024_dCas9 _seq_F_4	GTCGTTGGAACTGCTTTGATTA
0025_dCas9 _seq_F_5	GGAGCAGCATAAGCATTATTTAG
301-pTLR-sgYbeY1	CAATAATCTCACTTGGCACTGTTTAAGAGCTATGCTGGAAACAGCA
302-pTLR-sgYbeY2	TCTAGTGAGATAACATCTGTGTTTAAGAGCTATGCTGGAAACAGCA
303-pTLR-sgYbeY3	ACCATAGTCGGCAGCTTGTTGTTTAAGAGCTATGCTGGAAACAGCA
304-pTLR-sgYbeY4	AACATTTCTGACTCTTCTTCGTTTAAGAGCTATGCTGGAAACAGCA
305-PTLR-for all sgRNA	TAAACATATTATACTATTCCTACCCCACCTTAATATTTCT
306-gyrA-RT-Fw	TGTCAGTTATCGTTGCACGGG
307-gyrA-RT-Rv	TTCACCGACAATACGAGCCG
346-recover-Val-YbeY-Fw	ATGTACGTAGAATTAGTTGATGAAACAGGCCAAGTACCAAGTGAGATT ATTGAGC
347-recover-Val-YbeY-Rv	GCTCAATAATCTCACTTGGTACTTGGCCTGTTTCATCAACTAATTCTACG TACAT
348-recover-Pro-YbeY-Fw	ATGTACGTAGAATTAGTTGATGAAACAGGCCAAGTGCCCAGTGAGATT ATTGAGC
349-recover-Pro-YbeY-Rv	GCTCAATAATCTCACTGGGCACTTGGCCTGTTTCATCAACTAATTCTAC GTACAT
366-rt-mntH-Fw	TTGGTCAACGTCAATTACTGGAGGG
367-rt-mntH-Rv	AGAAGATATAAGGCAATAGCCGCACC
368-phoH-Fw	AGATTAATTATCGTGGGGAAATGGTGC
369-phoH-Rv	AGCAATGATAACATCCGACGCATGG
370-llmg1488-Fw	TGTGCCAATGGTAATAGCTTGTGC
371-llmg1488-Rv	TATACCCAGCTGACAAACCCACC
372-dgkA-Fw	TCGAGTTTAGAGTTTGCACTTACAGG
373-dgkA-Rv	AGCCATATCTTTAGCCCGTTTAGC
374-llmg1485-Fw	TGCAGGGAGTGTCTTTCAATCCGTC
375-llmg1485-Rv	AGGTGCATATAGGCTTCATTGTGG
391-phoH-promoter-Fw	TCCAACAGAGCCGCCTCATATGAGAATGCCAATGTGGGC
392-phoH-promoter-Rv	ATCGTGTGAGTCCATTAATCATTTTCTTACATTATAGCACATTTCATATTT TAATTTAAATCACA
393-ins-dgkA-llmg1485-Fw	GTAAGAAAATGATTAATGGACTCACACGATAAAGATGTAAAACTC

Primer name	Sequence (5' –> 3')
379-ins-dgkA-llmg1485-Rv	ACTAGTGCTCATTATGCTTTTTATCATTTATTAACTTTTAGTTCCGTCC
461-oe-ybey-ins-Fw	ACCACTAGTTCTAGAGAGCTCAAGCATGTACGTAGAATTAGTTGATGA AACAGGC
462-oe-ybey-ins-Rv	GCTATCAATCAAAGCAACACGTGCTTTATCGTGTGAGTCCATAGGCAGT
463-oe-ybey-back-Fw	GCTTGAGCTCTCTAGAACTAGTGGTACC
464-oe-ybey-back-Rv	AGCACGTGTTGCTTTGATTGATAGC
469-YbeyMU to dgkA to -backbone-Rv	TCTTCTGGCGTGTAATGATCGTAACC
470-YbeyMU to dgkA-backbone-Fw	AAGACTTAACGGCAGAGTGACGGGAGCAGAATGTCCGAGACTA
471-YbeyMU to dgka-ins-Fw	TTACGATCATTACACGCCAGAAGAAGAGTC
472-YbeyMU to dgka-ins-Rv	TCCCGTCACTCTGCCGTTAAGTCTTCACTTAGATC
n273-argG-RT-Fw	ATGGATGTTGGTGAAGGGAAGGAC
n274-argG-RT-Rv	AGCACCAACGAAGATTTCAGCG
n275-argC-RT-Fw	AGTCAGTCAAGCCTTCAAGTTGC
n276-argC-RT-Rv	ATAGCGGCTTAGAACTGTTCCG
n277-arcA-RT-Fw	ACATTGCGTGACAATGGTGCTG
n278-arcA-RT-Rv	AGTCAATCCATCATAAGTACGTCCTGG
n273-argG-RT-Fw	ATGGATGTTGGTGAAGGGAAGGAC
n266-sgArgX-Fw	GAGTGTGACTTGAATCAAAGTTTAAGAGCTATGCTGGAAACAGCA

Table S2 sRNAs in *L. lactis* can be targeted with sgRNAs.

sRNA	sequence	PAM	IGR/3'-UTR	Verified
LLMGnc_002	ATCGGCCTTTTGGCTTTTATTGAAATAT	1	IGR	
LLMGnc_005	AGCGTCCTATTTCAAACTAGGGCGCTTT	1	IGR	
LLMGnc_008	GAGATCCTCTTGCTTATGGTGCCGGGGTGAGC AGTGAGAAAACT	2	IGR	
LLMGnc_009	TCATACCTGTCCAATTTGGACTAAGGTT	1	IGR	
LLMGnc_010	TTGTACCTGGATATGTGCCAAAACTATC	1	IGR	yes
LLMGnc_016	TCTTGCCATCTTATTGACTAGGTGAACA	1	IGR	
LLMGnc_030	TGAAGCCTTTAGTCTGACAGAGTTCACT	1	IGR	
LLMGnc_035	CGTCCTCCTAGAAACTAGGAAAGTAG--- CGGAACCTGCACAAGTGCAGAACCACGATTC CCTACCTTACGTTGTCCGCTCTCGCTA	5	IGR	
LLMGnc_036	TCTTGCCAGCTTTGCTGGCTTAGAACGA	1	IGR	
LLMGnc_039	TTGGTCCGATAGCTCAGCTGGCTCGAAG	1	3'-UTR of *lytR*	
LLMGnc_041	TACGACCAAGCCCCAAACTCACATCATA	1	3'-UTR of *llmg_0573*	
LLMGnc_047	TATCTCCAAGGGTGAGGAGATTGTTTTG	1	IGR	
LLMGnc_051	GGTTTCCTACTGTTAGAAGTCAGACGAT	1	IGR	
LLMGnc_054	GGACACCAGAGTATACTCCAATGTCATT	1	IGR	
LLMGnc_064	GTTTTCCGCGCAGGAAGCGTGAGCATTT	1	IGR	yes
LLMGnc_081	TTACACCACATGACGGCTGAATTGCAAC	1	IGR	
LLMGnc_083	AGAAACCAGTTCGGTATGATCGTCTATG	1	IGR	
LLMGnc_087	GACTCCTATGACACTGAAGGTCTATAA	1	IGR	yes
LLMGnc_096	AATGACCTCATCAGCTTGGGTACATTAA	1	IGR	
LLMGnc_100	AAAATCCATGTCATTACGATATGGATTT	1	IGR	
LLMGnc_102	CGGTACCTGCCGATTAAGAACTCCATACGTTC	2	IGR	

LLMGnc_103	AGGTACCGATGAAGTTTGCGCTTCAAAG	1	IGR	
LLMGnc_106	TTTGACCGCTCAAGATTACGGATACTTT	1	IGR	
LLMGnc_112	GGATACCCCATAGCGTTCGTGAGTAAAA	1	IGR	
LLMGnc_117	GACTTCCTGTAATGTGGAGTATACACCACTCT GTCTCACCACGGTGTA	2	IGR	
LLMGnc_120	AAAATCCACTTAATAGACGGAACAGTCG	1	IGR	
LLMGnc_126	AAAAACCATCCAGCTTGGACAGTAGATA	1	IGR	
LLMGnc_134	AATAATCCTGCCCTCTCTTGGATTCATTGCTAT	2	IGR	
LLMGnc_138	ACAGACCGCGTGTTCAAACTTGGTTATG	1	IGR	yes
LLMGnc_145	CAAAACCATCAGCAACTGCTGGAAGAAC	1	IGR	
LLMGnc_147	GTTTACCTTTAAGTCATGGTTACGTTAC	1	IGR	
LLMGnc_152	ATCATCCTCTAGTTGGACTCCTTGAAGTCATC CAGCTAGAGC	2	IGR	
LLMGnc_153	TTCATCCTATTTCTAGAGTTAAGCTGCT	1	IGR	
LLMGnc_157	TTTATCCGAAGCGTCCCTCTCCTAACTG	1	IGR	
LLMGnc_164	AAATACCACTCAACCTTACAAGCCTGAC	1	IGR	
LLMGnc_169	TTATTCCACCTAGATAGAGGTGGGGAAG	1	IGR	
LLMGnc_170	AAGAACCAATGGAAAGTTAAGAAAGTTC	1	IGR	
LLMGnc_172	ATAAACCCCTTTGATTCAAGTCACACTC	1	3'-UTR of *argR*	yes
LLMGnc_177	AAACTCCTTGAAACTCTCGTTCTTATGA	1	3'-UTR of *zitRS*	yes
LLMGnc_180	AGCAACCACTAATTTGGTTGCTCCTTTT	1	IGR	
LLMGnc_182	ACAAGCCTGACAGGGCGATTCGGCTCTT	1	IGR	
LLMGnc_184	CTCCCCCGTATCTTTTCGCTGGGAAAGC	1	IGR	yes
